# Enhanced Interfacial Contact and Lithium-Ion Transport in Ionic Liquid Polymer Electrolyte via In-Situ Electrolyte-Cathode Integration

**DOI:** 10.3390/molecules30020395

**Published:** 2025-01-18

**Authors:** Zehua Chen, Mianrui Li, Shengguang Qi, Li Du

**Affiliations:** Guangdong Provincial Key Laboratory of Fuel Cell Technology, School of Chemistry and Chemical Engineering, South China University of Technology, Guangzhou 510641, China; chenzh48944@163.com (Z.C.); 202410186301@mail.scut.edu.cn (M.L.); samuel_qsg@aliyun.com (S.Q.)

**Keywords:** integrated electrolyte/cathode structure, in situ UV photopolymerization, composite solid electrolyte, lithium metal batteries, ionic liquid

## Abstract

Solid polymer electrolytes (SPEs) have attracted much attention due to their excellent flexibility, strong interfacial adhesion, and good processibility. However, the poor interfacial contact between the separate solid polymer electrolytes and electrodes leads to large interfacial impedance and, thus, hinders Li transport. In this work, an ionic liquid-modified comb-like crosslinked network composite solid-state electrolyte with an integrated electrolyte/cathode structure is prepared by in situ ultraviolet (UV) photopolymerization. Combining the enhanced interfacial contact and the introduction of ionic liquid, a continuous and fast Li^+^ transport channel at the electrolyte–cathode interface is established, ultimately enhancing the overall performance of solid-state lithium batteries. The composite solid electrolytes (CSEs) exhibit an ionic conductivity of 0.44 mS cm^−1^ at 60 °C. LiFePO_4_//Li cells deliver a high discharge capacity (154 mAh g^−1^ at 0.5 C) and cycling stability (with a retention rate of more than 80% at 0.5 C after 200 cycles) at 60 °C.

## 1. Introduction

Lithium-ion batteries (LIBs) are now widely used as efficient energy storage devices to fulfill ever-increasing demands for various application scenarios. Due to the exceptionally high theoretical specific capacity of lithium metal anode (3860 mAh g^−1^), which surpasses that of graphite anode (372 mAh g^−1^), lithium metal batteries (LMBs) are regarded as a new generation of high energy density lithium battery systems. Nevertheless, the organic liquid electrolytes used in LMBs are toxic, flammable, volatile, and thermodynamically unstable to lithium metal, leading to serious safety hazards. Solid-state lithium batteries (SSLBs) are the most promising candidates for the next-generation energy storage devices, in which solid-state electrolytes (SSEs) replace traditional separators and liquid electrolytes. As a type of SSEs, SPEs are considered an ideal solution for the next generation of SSLBs due to their excellent flexibility, lightweight, low processing costs, and compatibility with contemporary roll-to-roll fabrication processes for LIBs. These characteristics contribute to achieving high specific energy, enhanced safety, and long cycle life.

The interfacial contacts between electrolyte/electrode vary significantly between traditional liquid LIBs and SSLBs. In traditional liquid LIBs, electrolytes effectively wet the electrode surface, whereas, in SSLBs, the SSEs are in rigid contact with the electrode. This difference results in elevated interfacial resistances in SSLBs. Therefore, reducing the interfacial resistance between the SSEs and the electrode is crucial for the development of SSLBs. Currently, the traditional preparation methods of SPEs are generally independent of electrodes, such as solution casting and electrospinning. The ex situ methods inevitably lead to poor interfacial contact at the electrolyte–cathode interface, unfavorable to Li transport. Moreover, these methods exhibit low preparation efficiency. The solution casting requires a long time to ensure complete solvent evaporation. Furthermore, electrospinning typically has a slow production efficiency. In contrast, solventless in situ UV polymerization is a promising method for the rapid fabrication of SPEs due to its high reaction productivity. Hence, the SPEs with electrolyte–electrode integration via in situ UV polymerization are expected to reduce interfacial impedance and realize the rapid synthesis of the electrolytes [1,2,3,4,5,6,7,8].

The low ionic conductivity is another obstacle that restricts the application of SPEs. Researchers have shown that garnet-type oxide solid electrolytes can be used as inorganic fillers to prepare enhanced mechanical strength and high ionic conductivity CSEs [9,10,11]. However, the ceramic fillers bring about dilution and a blocking effect, leading to severe agglomeration and the destruction of conducting pathways, impeding the transport of Li and decreasing the ionic conductivity of CSEs. To tackle the issue, ionic liquids (ILs) have been adopted in CSEs to create new transport pathways for Li through ceramic fillers and the polymer matrix [12,13,14,15,16,17]. ILs composed of self-dissociated cations and anions are low-temperature or room-temperature molten salts that wet interfaces and act as fast ionic conductors [18,19,20]. In particular, N-butyl-N-methylpyrrolidinium bis(trifluoromethanesulfonyl)imide (Pyr_14_TFSI) shows a wide electrochemical stability window and lower viscosity, significantly wetting the electrode and reducing contact impedance. In such CSEs, Li^+^ transference could be accelerated through various pathways in the polymer matrix, ceramic fillers, ceramic-polymer interface, and IL-modified phases of the polymer matrix.

Herein, by incorporating garnet-type Li_6.5_La_3_Zr_1.5_Ta_0.5_O_12_ (LLZTO) nanopowder as inorganic fillers and nonflammable ILs as fast ionic conductors, we designed pyrrolidinium-type ILs-modified CSEs with electrolyte–cathode integrated structure via in situ UV-initiated polymerization. Attributed to the multiple Li^+^ transport channels derived from ILs, the CSEs exhibit a high ionic conductivity of 0.44 mS cm^−1^ at 60 °C. LiFePO_4_//Li cells with this electrolyte deliver a high discharge capacity (154 mAh g^−1^ at 0.5 C) and cycling stability (with a retention rate of more than 80% at 0.5 C after 200 cycles) at 60 °C. In addition, the electrolyte–cathode integration through in situ UV polymerization significantly reduces the impedance of CSEs (70 Ω cm^2^) than that of ex situ structure (125 Ω cm^2^). The fabrication method not only simplifies the preparation process but also enhances the interfacial contact. This work may provide new insights into the advancement of efficient SSLBs from the perspective of both processibility and multiple ion conductive channel design.

## 2. Results and Discussion

### 2.1. Design and Preparation of Electrolytes

**Figure 1:**
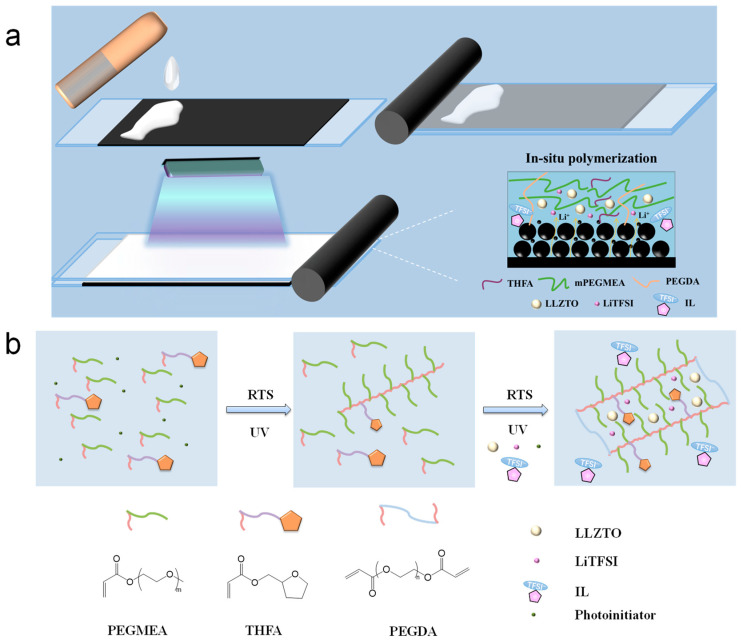
(**a**) Schematic diagram of the preparation process of electrolyte–cathode integrated ionic liquid gel electrolyte; (**b**) schematic diagram of polymer molecule structure.

The preparation process of the electrolyte–cathode integrated ionic liquid gel electrolyte by blade coating combined with in situ UV polymerization is illustrated in Figure 1a. Equipped with shear thinning and tunable viscosity properties, the resulting slurry can be directly coated onto the cathode electrodes and then cured into CSEs under a UV lamp, which can realize facile and scalable CSEs fabrication and construct a continuous and fast Li^+^ transport channel. Figure 1b illustrates the polymer molecule structure synthesized by the two-stage solventless UV polymerization strategy. Poly(ethylene glycol) acrylates (PEGAs) are a promising polymer matrix for SPEs due to their low crystallization and high lithium salt dissociation ability. PEGAs are able to realize rapid UV-induced gelation in the presence of a photoinitiator, which features efficient and rapid fabrication of SPEs [21,22,23,24,25,26]. In order to optimize Li^+^ transport channels, tetrahydrofurfuryl acrylate (THFA) is utilized to copolymerize with PEGAs for polymer molecule structure design. The cyclic tetrahydrofurfuryl active group in THFA tends to bond with Li^+^ more effectively than the C–O–C group of the PEG side chain, which helps to disrupt the ordered structure of polymer chain segments and decrease the glass transition temperature (*T_g_*) [26]. Firstly, to prepare the electrolyte, poly(ethylene glycol) methyl ether acrylate (PEGMEA), THFA, and photoinitiators are mixed. Part of the monomers undergo C=C double bonds polycondensation reactions under the 1st UV-initiated polymerization; the mixture converts to viscous rheology-tuning slurry with THFA (RTS-TH) slurry. After rheology-tuning, the RTS-TH slurry is evenly mixed with polyethylene glycol diacrylate (PEGDA), lithium bis(trifluoromethanesulphonyl)imide (LiTFSI), Pyr_14_TFSI, LLZTO and additional photoinitiators, fully polymerized and finally cured into ILs-modified CSEs with a comb-like crosslinked network structure under the 2nd UV-initiated polymerization.

### 2.2. Physicochemical Characterization of CSEs

**Figure 2:**
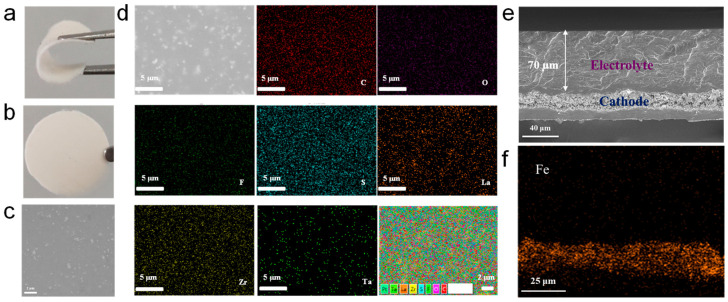
(**a**,**b**) Digital photograph of free-standing RTS-TH-IL CSE; (**c**) SEM image of free-standing RTS-TH-IL CSE surface; (**d**) EDS mapping image of C, O, F, S, La, Zr and Ta elements in CSE; (**e**) Cross-section SEM image of in situ synthesized RTS-TH-IL CSE on LiFePO_4_; (**f**) Corresponding EDS mapping image of the distribution of Fe element.

The digital photograph of free-standing RTS-TH CSE with IL (RTS-TH-IL CSE) is presented in Figure 2a,b. The CSEs not only exhibit a flat, smooth, and compact surface without any cracks, but they also possess excellent bend-resistant characteristics. Figure 2c demonstrates that the LLZTO is uniformly distributed with no obvious agglomeration. Furthermore, the energy dispersive spectrometer (EDS) mapping images are shown in Figure 2d. The C, O, F, S, La, Zr, and Ta elements in RTS-TH-IL CSE are evenly distributed, further indicating that LLZTO, LiTFSI, and Pyr_14_TFSI are uniformly dispersed within the CSEs. As shown in Figure 2e,f, the cross-sectional scanning electron microscope (SEM) image and the EDS mapping image of the in situ synthesized RTS-TH-IL CSE on LiFePO_4_ were obtained to verify and evaluate the interface between the cathode and the electrolyte. The electrolyte–cathode integrated structure can provide a tight, effective, and void-free interfacial contact, which eliminates separated and rough interfaces, constructing a well-connected and continuous Li^+^ transport channel, and remarkably reduces the interface impedance. The cross-section SEM image reveals that the final thickness of blade-coated in situ RTS-TH-IL CSE is about 70 μm. Compared to some free-standing CSEs with thickness above 100 μm, these thinner CSEs have a shorter Li^+^ diffusion distance and smaller mass and volume, which can be favorable for improving the comprehensive electrochemical performance and reducing the overall cost [27].

**Figure 3:**
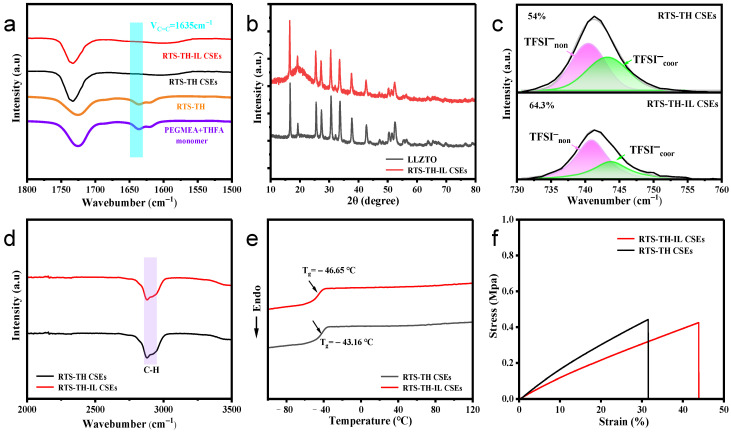
(**a**) FTIR spectra of PEGMEA/THFA monomer, RTS-TH, RTS-TH CSE, and RTS-TH-IL CSE; (**b**) XRD spectra of LLZTO powder and RTS-TH-IL CSE; (**c**) Raman spectra of TFSI anion in RTS-TH CSE and RTS-TH-IL CSE; (**d**) FTIR spectra of RTS-TH CSE and RTS-TH-IL CSE; (**e**) DSC curves of RTS-TH CSE and RTS-TH-IL CSE; (**f**) Stress–strain curves of RTS-TH-IL CSE and RTS-TH CSE.

In the Fourier transform infrared (FTIR) spectra of Figure 3a, the peak at 1635 cm^−1^ corresponds to the adsorption of the C=C double bond vibration. After part of the PEGMEA and THFA monomers underwent polycondensation reaction, we can see that the adsorption peak at 1635 cm^−1^ of RTS-TH still exists, but the intensity of this peak is slightly weaker than that of monomers. Under the 2nd UV-initiated polymerization, the adsorption peak at 1635 cm^−1^ of RTS-TH-IL CSE and RTS-TH CSE without IL (RTS-TH CSE) disappears, which indicates that the fully polymerized CSEs with crosslinked networks are obtained. In this architecture, a comb-like crosslinked molecular network structure of CSEs with oligoethylene oxide pendants swinging freely is established. The X-ray diffractometer (XRD) data in Figure 3b shows that the cubic garnet crystalline phase of LLZTO remains stable in RTS-TH-IL CSE, indicating that the addition of ILs does not destroy the LLZTO structure. The interaction between Li^+^ and TFSI^−^ in the RTS-TH-IL CSE and RTS-TH CSE was further explored by Raman spectroscopy of Figure 3c. The characteristic peaks in a sharp range of 740 cm^−1^−744 cm^−1^ can be used to distinguish free state TFSI^−^ (TFSI^−^_non_) at 740 cm^−1^−741 cm^−1^ and the bound state [Li^+^−TFSI^−^] or the cluster state [Li (TFSI^−^)_2_] ^−^ (TFSI^−^_coor_) at 743 cm^−1^−744 cm^−1^. According to $x=A_{\mathrm{non}}/(A_{\mathrm{non}}+A_{\mathrm{coor}})$, in which x represents the proportion of the TFSI^−^_non_ region that occupies the region of all LiTFSI existence forms, the percentage of TFSI^−^_non_ is 64.3% with ILs and 54% without ILs. The increase in the TFSI^−^_non_ and the decrease in the TFSI^−^_coor_ indicate that TFSI^−^ is released from the bound state or cluster state, further demonstrating that the addition of ILs facilitates the dissociation of LiTFSI. Studies show that TFSI^−^_coor_ can hinder Li^+^ conducting, while the increase in free Li^+^ contributes to improving Li^+^ carrying capacity, the ionic conductivity, and the Li^+^ transference number [28].

As shown in the FTIR spectra of Figure 3d, the intensity of the C-H stretching characteristic peak in RTS-TH-IL CSE is weakened, which indicates that the addition of ILs is beneficial to reducing the crystallinity of the CSEs [29]. In the Differential Scanning Calorimetry (DSC) curves of Figure 3e, the T_g_ values of RTS-TH CSE and RTS-TH-IL CSE are −43.2 °C and −46.7 °C, respectively, indicating that the addition of ILs effectively reduces the crystallinity, provides more free volume of the amorphous region, and promotes the chain segment movement. As shown in the stress–strain curves of Figure 3f, after adding the ILs, the elongation at break of CSEs is increased from 31% to 43%, while the tensile strength of the CSEs decreases slightly but remains at 0.4 MPa. Thus, the results indicate that the addition of ILs can enhance the mechanical performance of the CSEs, which contributes to improving the flexibility and deformation capability of the CSEs and reduces the possibility of structural fracture and lithium dendrite penetration.

### 2.3. Electrochemical Behaviors of CSEs

**Figure 4:**
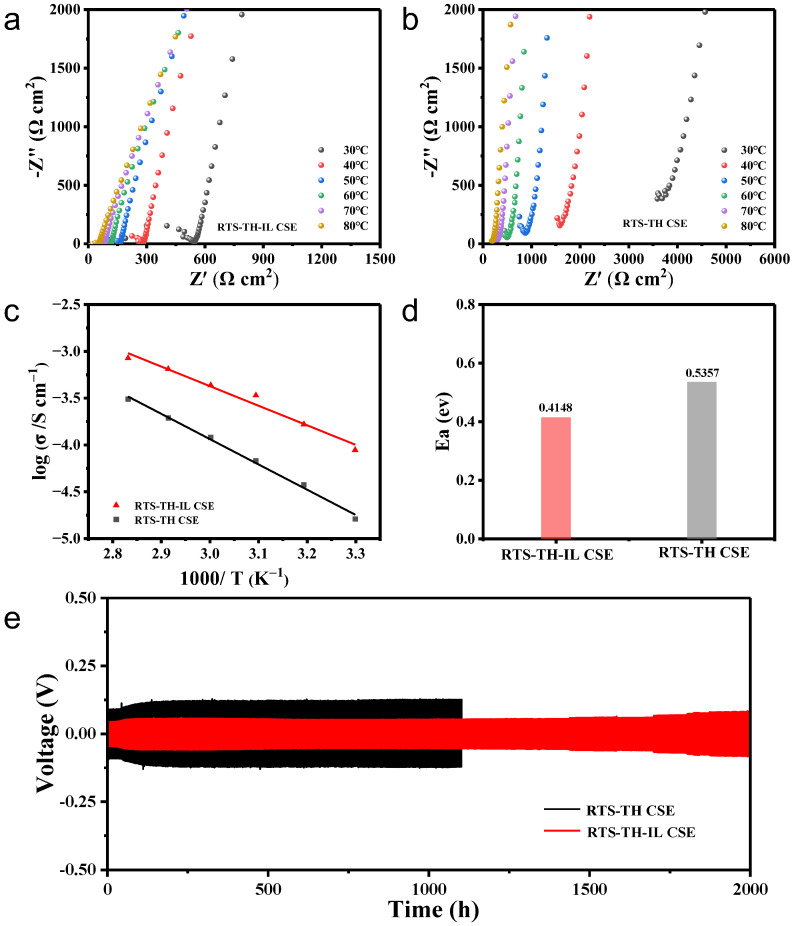
Nyquist curves in the temperature range from 30 °C to 80 °C of (**a**) RTS-TH-ILCSE and (**b**) RTS-TH CSE; (**c**) Arrhenius plots of ionic conductivities of RTS-TH-IL CSE and RTS-TH CSE; (**d**) activation energy of RTS-TH-IL CSE and RTS-TH CSE; (**e**) galvanostatic charge–discharge cycling curves of Li//Li symmetric cells with RTS-TH CSE and RTS-TH-IL CSE at 60 °C at 0.1 mA cm^−2^ @ 0.1 mAh cm^−2^.

The Nyquist curves of CSEs in Figure 4a,b show that RTS-TH-IL CSE exhibit higher ionic conductivity than RTS-TH CSE at all temperatures. From Tables S1 and S2, the ionic conductivities of RTS-TH-IL CSE are 7.8 × 10^−5^ S cm^−1^ at 30 °C and 4.37 × 10^−4^ S cm^−1^ at 60 °C, respectively, which are approximately 3.7 times higher than those of RTS-TH CSE (1.6 × 10^−5^ S cm^−1^ at 30 °C and 1.2 × 10^−4^ S cm^−1^ at 60 °C). On the one hand, the porosity and the interaction of the PEGAs/LLZTO network can act as a host for the permeation and retention of ILs so as to form a continuous and rapid Li^+^ diffusion pathway, which significantly enhances ionic conductivity [27]. On the other hand, ILs can wet the interface to reduce the interfacial impedance. According to the Arrhenius plots of CSEs shown in Figure 4c and the Arrhenius equation σ (T) = A exp (−E_a_/RT), the activation energies (E_a_) for RTS-TH-IL CSE and RTS-TH CSE calculated in Figure 4d are 0.4148 eV and 0.5357 eV, respectively, indicating a lower diffusion barrier for Li^+^ migration in the RTS-TH-IL CSE. The cycling profiles of Li//Li symmetric cells with CSEs were tested at 0.1 mA cm^−2^ @ 0.1 mAh cm^−2^, as shown in Figure 4e, indicating that the Li/RTS-TH-IL CSE/Li cells exhibit excellent electrochemical performance with an overpotential of under 60 mV and stable cycling for over 2000 h without short circuits. This indicates the stable interface during the Li deposition and stripping processes. In addition, the overpotential of Li/RTS-TH-IL CSE/Li cells is lower than that of RTS-TH CSE, which can be attributed to the enhanced ionic conductivity of the CSEs. Therefore, the addition of ILs inhibits dendrite growth and builds a continuous and rapid Li^+^ diffusion pathway. The critical current density (CCD) test of CSEs in Figure S4b demonstrates that Li/RTS-TH-IL CSE/Li cells keep stable cycling at 0.8 mA cm^−2^, while the overpotential of RTS-TH CSE increases dramatically at 0.6 mA cm^−2^. The increased CCD indicates that the solid electrolyte interface (SEI) between the lithium metal and the RTS-TH-IL CSE is highly stable, and there are no additional side reactions with ILs. Moreover, Figure S5 indicates that the Li^+^ transference number (t_Li_^+^) of RTS-TH-IL CSE is about 0.22, higher than that of RTS-TH-CSE (t_Li_^+^ = 0.10), benefiting for alleviating the concentration polarization inside the battery and providing a uniform Li^+^ flux for even Li plating and stripping. In addition, the introduction of LLZTO can promote the dissociation of TFSI^−^ from ILs. The adsorption energy of Pyrr^+^ on the surface of inorganic particles is lower than that of TFSI^−^. The results indicate that Pyrr^+^ is prone to absorb onto the surface of LLZTO, contributing to the dissociation of the TFSI^−^ and Pyrr^+^. Studies show that the free state TFSI^−^ form ILs and LiTFSI migrating to anode primarily undergoes the transformation of a twenty-electrons reductive decomposition reaction ((CF_3_SO_2_)_2_N^−^ + 21 Li^+^ + 20 e^−^→Li_3_N + 2 Li_2_S + 4 Li_2_O + 6 LiF) and eventually generates the LiF-rich SEI layer at the anode interface, which facilitates the uniform deposition of Li^+^ for inhibition of growth of lithium dendrites to prolong the battery cycling and improves the electrochemical stability of CSEs [30].

### 2.4. Electrochemical Performance of CSE-Based Cells

**Figure 5:**
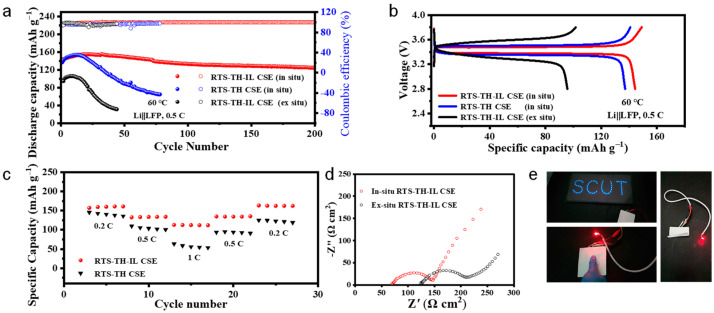
(**a**) Cycling performance and coulombic efficiency of LiFePO_4_//Li cells in the voltage range of 2.5 V to 3.8 V at 0.5 C at 60 °C. (**b**) Initial galvanostatic charge/discharge curves of LiFePO_4_//Li cells at 0.5 C at 60 °C; (**c**) rate capability of LiFePO_4_//Li cells; (**d**) EIS of LiFePO_4_//Li cells comparing the impedance with in situ RTS-TH-IL CSE and ex situ RTS-TH-IL CSE; (**e**) a red LED and the blue LED light row powered by the LiFePO_4_//Li pouch cells employing in situ RTS-TH-IL CSE.

The cycling stability of the Li/CSEs/LiFePO_4_ cells at 0.5 C in Figure 5a and the corresponding charge/discharge curves of different cycles in Figure S6 shows that the cell adopting ex situ RTS-TH-IL CSE displays a low initial discharge capacity of 101 mAh g^−1^ and experiences rapid capacity degradation up until the 20th cycle. In addition, the cell with in situ RTS-TH CSE shows a slightly higher initial discharge capacity due to the improved interfacial contact but still displays rapid capacity degradation until the 20th cycle because of low Li^+^ mobility. Comparatively, the cells with in situ RTS-TH-IL CSE exhibit an initial discharge capacity of 144 mAh g^−1^ and can reach the highest capacity of 154 mAh g^−1^ after the activation process during the first few cycles. Furthermore, the specific capacity of the cells employing in situ RTS-TH-IL CSE retains at 125.1 mAh g^−1^ after 200 cycles, and the capacity retentions at 100th and 200th cycles are 90% and 80%, indicating that the synergistic effect of in situ UV polymerization and the introduction of ILs can effectively improve the performance of cells.

Compared to in situ RTS-TH CSE and ex situ RTS-TH-IL CSE, the initial charge/discharge voltage profiles of Li/CSEs/LiFePO_4_ cells in Figure 5b show that the cell with in situ RTS-TH-IL CSE exhibits a higher specific capacity and smaller polarization voltage due to the continuous and fast Li^+^ transport channel and sufficient utilization of active materials. In addition, the cell with in situ RTS-TH CSE exhibits a smaller polarization voltage than that with ex situ RTS-TH-IL CSE, indicating that the electrolyte–cathode interfacial contact is a critical factor for the electrochemical performance of cells.

Figure 5c shows that the rate capability of the cells employing in situ RTS-TH-IL CSE is highly improved, and the discharge capacities are 157, 132.5, and 116.4 mAh g^−1^ at 0.2, 0.5, and 1 C, respectively. Moreover, the discharge-specific capacity can return to its original value along with the return of current densities, indicating better cycling stability and reversibility. The electrochemical impedance spectroscopy (EIS) results in Figure 5d reveal that the bulk resistance of the cells with in situ RTS-TH-IL CSE is approximately 56% of that of the cells with ex situ RTS-TH-IL CSE (70 Ω cm^2^ vs. 125 Ω cm^2^), which is consistent with the blade-coatable film being significantly thinner (75 μm vs. 150 μm). Moreover, the interfacial resistance of the cells with in situ RTS-TH-IL CSE also yields a 32.7% lower interfacial impedance than that with ex situ RTS-TH-IL CSE (142 Ω cm^2^ vs. 211 Ω cm^2^), resulting in a lower overall cell impedance for higher energy density and better cycling stability. In addition, the impedance of CSEs can be decreased by reducing the thickness. It is found that the in situ polymerization can fabricate thinner and more conformable CSEs to meet the demand for bending and adapting better to deformation, while the ex situ CSEs need to achieve a self-standing thickness for assembly and may be more likely to delaminate from the electrodes under uneven forces, which can severely degrade battery performance. Furthermore, ILs may penetrate from CSEs into the porous electrodes during the battery operation, benefiting from wetting the electrodes and accelerating the mass transport and charge transfer kinetics [31]. Due to the high viscosity property of ILs, IL-modified CSEs with the electrolyte/cathode integrated structure can construct a viscoelastic interface between the electrode and the electrolyte, and provide sufficient penetration and diffusion pathways for Li^+^, which can significantly mitigate the interfacial and overall cell impedance and accommodate the large volume changes in cathode during cycling. Figure 5e illustrates that the pouch cell, which is paired together with in situ RTS-TH-IL CSE and a 250 μm lithium foil wrapped in an Al-plastic film, effectively powers up a red LED bulb and a blue LED light strip both before and after it has been folded. This demonstrates the flexibility and safety of the obtained CSEs.

## 3. Materials and Methods

### 3.1. Preparation of RTS-TH

PEGMEA (M_w_ = 518, Sartomer CD551, 45 g), THFA (5 g), and 2,2-dimethoxy-2-phenylacetophenone photoinitiator (0.03 g) were added into a flask filled with nitrogen. After mixing evenly, the solution was exposed to a 365 nm UV lamp for several minutes until a viscous slurry formed. Meanwhile, the UV lamp and nitrogen were removed to stop the reaction.

### 3.2. Preparation of RTS-TH-IL CSE and RTS-TH CSE

For the RTS-TH-IL slurry, RTS-TH slurry (2.1 g), and PEGDA (Mw = 608, Sartomer SR610, 0.422 g) were added into a brown glass bottle for the preliminary mix. Next, Pyr_14_TFSI (Shanghai Chengjie Chemical Co., Shanghai, China, 99%, 0.422 g), LiTFSI (0.856 g), LLZTO (500 nm, Shenzhen Kejing Zhida Co., Ltd., Shenzhen, China, 0.422 g), and a photoinitiator (0.04 g) were added sequentially. For the RTS-TH slurry, RTS-TH slurry (2.4 g) and PEGDA (0.422 g) were added in a brown glass bottle for preliminary mix. Then, LiTFSI (0.978 g), LLZTO (0.422 g), and the photoinitiator (0.04 g) were added in sequence. The mixture was stirred for several hours until the LLZTO was fully dispersed. The free-standing CSEs were prepared by blade-coatable method with the above slurry, followed by UV curing at 365 nm for 10 min to obtain a thickness of 250 μm for the RTS-TH-IL CSE or RTS-TH CSE.

### 3.3. Preparation of LiFePO_4_ Cathode

The LiFePO_4_ cathode was fabricated by dissolving LiFePO_4_ (active material, 70 wt%), Poly (vinylidene fluoride) (PVDF, 10 wt%), Poly (ethylene oxide), (PEO, M_w_= 600,000, Macklin., 10 wt%), and Super P (10 wt%) in N-methyl-2-pyrroldone (NMP). After mixing evenly, the slurry was coated on carbon-coated Al foil (C-Al, 18 μm, Hefei Kejing Material Technology Co., Hefei, China). The coated cathode was then placed in a vacuum-drying oven and dried at 60 °C overnight. Finally, the dried cathode was punched into pellets with a diameter of 12 mm, achieving a mass loading of 1 mg cm^−2^.

### 3.4. Preparation of CSEs with Electrolyte-Cathode Integrated Structure by In Situ UV Polymerization

Using the blade coating, the RTS-TH/RTS-TH-IL slurry was coated on the cathode electrode, and then cured with a UV light at 365 nm for 10 min to obtain RTS-TH/RTS-TH-IL CSE with a thickness of about 70 μm.

### 3.5. Physical Characterization

The MERLIN SEM was used to observe the morphologies of CSEs. The MiniFlex (Rigaku, Tokyo, Japan) XRD was used for the crystal phase test with Cu Kα radiation scanning from 10° to 80° at the rate of 5° min^−1^. FTIR spectra using NicoletIS50 (Thermo Fisher Scientific, Waltham, MA, USA) were utilized to analyze the composition of the sample. DSC was conducted to determine the T_g_ of the CSEs on the Polyma 214 instrument. The mechanical properties were evaluated using the Instron 5967 tensile test machine (Instron, Norwood, MA, USA). Raman spectra were obtained on a LabRAM HR Evolution Raman spectrophotometer (HORIBA, Kyoto, Japan).

### 3.6. Electrochemical Characterization

The Autolab Electrochemical Instrumentation (Metrohm, Herisau, Switzerland) was used to evaluate the electrochemical properties. EIS of the C-Al/CSE/C-Al blocking cell was used to evaluate the ionic conductivities (σ) of the CSEs in the frequency range from 100 kHz to 0.1 Hz. The σ can be calculated by the following equation:

$$\sigma =\frac{L}{RA}$$

where *L* is the thickness of the CSE membrane, *R* represents the bulk resistance obtained from alternating current impedance analysis, and *A* is the contact area between C-Al and the electrolyte.

Li^+^ transference number (*t_Li_*^+^) was calculated by the following equation:

$$t_{{Li}^{+}}=\frac{I_{ap}(∆V-I_{bp}R_{bp})}{I_{bp}(∆V-I_{ap}R_{ap})}$$

The initial current (*I_ap_*) and the steady current (*I_bp_*) of the Li/CSE/Li symmetric cell were measured with a voltage pulse (Δ*V*) of 10 mV in DC polarization mode. *R_bp_* and *R_ap_* represent the resistance before and after polarization, respectively. To obtain Li//Li symmetric cells, the CSEs were sandwiched with two Li foils (diameter = 8 mm) in CR2025-type coin cells. To do the cell assembly, a CR2025-type coin cell was assembled by contacting, in sequence, the in situ CSEs punched by multi-punch pliers (d = 12 mm), an 8 mm concentric pore PP Separator (Celgard., Charlotte, NC, USA, 25 μm), and a Li foil (d = 10 mm). All batteries were assembled in an argon-filled glove box (H_2_O < 0.1 ppm, O_2_ < 0.1 ppm). On a LAND-CT3001A battery tester (Wuhan LAND Electronic Co., Ltd., Wuhan, China), galvanostatic charge/discharge measurements were conducted to evaluate the cyclic performance of Li//Li symmetric cells and LiFePO_4_//Li cells. The LiFePO_4_//Li cells were tested at a voltage range of 2.5 V−3.8 V. The electrochemical measurement tests described above were all conducted at 60 °C.

## 4. Conclusions

In summary, IL-modified CSEs with electrolyte-cathode integrated structures are fabricated via in situ UV-initiated polymerization. The RTS-TH-IL CSEs exhibit a maximum ionic conductivity of 0.437 mS cm^−1^ at 60 °C. The addition of ILs facilitates the dissociation of LiTFSI and reduces the crystallinity of CSEs, which can provide more free volume in the amorphous region, effectively improving the ionic conductivity and Li^+^ mobility number. The synergistic effect of in situ UV polymerization and the introduction of ILs can provide sufficient penetration and diffusion pathways for Li^+^, significantly decreasing the interfacial resistance of SSLBs and improving the performance of cells. The Li//Li symmetric cells with the RTS-TH-IL CSE cycle remained stable for over 2000 h. LiFePO_4_//Li cells employing RTS-TH-IL CSE exhibit impressive cycling stability, with a discharge capacity of 125.1 mAh g^−1^ and more than 80% retention after 200 cycles at 0.5 C. The solventless UV polymerization strategy holds great promise for realizing the rapid, facile, and large-scale fabrication of CSEs. This study may offer new insights into the development of efficient ionic liquid solid-state lithium batteries, emphasizing both processability and the design of multiple ion conductive channels.

## Data Availability

The data presented in this study are available on request from the corresponding authors.

## Supplementary Materials

The following supporting information can be downloaded at: https://www.mdpi.com/article/10.3390/molecules30020395/s1, Figure S1: (a) Galvanostatic charge-discharge cycling curves of Li//Li symmetric cells with different LLZTO mass ratio in RTS-TH-LiTFSI-PEGDA-XLLZTO at 60 °C at 0.1 mA cm^−2^ @ 0.1 mAh cm^−2^; (b) Zoom-in curves at 270 h–276 h in (a); (c) Zoom-in curves at 468 h–474 h in (a); Figure S2: (a) Galvanostatic charge-discharge cycling curves of Li//Li symmetric cells with different EO:Li^+^ ratio in RTS-TH-LLZTO10-PEGDA-LiTFSI_x_ at 60 °C at 0.1 mA cm^−2^ @ 0.1 mAh cm^−2^; (b) Zoom-in curves at 250 h–258 h in (a); (c) Zoom-in curves at 994 h–1000 h in (a); Figure S3: (a) Nyquist curves of RTS-TH-LLZTOx-PEGDA-LiTFSI_6_ at 30 °C; (b) Nyquist curves of RTS-TH-LLZTO10-PEGDA-LiTFSI_x_ at 30 °C; (c) Galvanostatic charge-discharge cycling curves of Li//Li symmetric cells with RTS-TH-PEGDA-LiTFSI_6_ at 60 °C at 0.1 mA cm^−2^ @ 0.1 mAh cm^−2^; (d) Galvanostatic charge-discharge cycling curves of Li//Li symmetric cells with RTS-TH-LLZTO10-PEGDA-LiTFSI_20_ at 60 °C at 0.1 mA cm^−2^ @ 0.1 mAh cm^−2^; Figure S4: Galvanostatic charge-discharge cycling curves of Li//Li symmetric cells with RTS-TH CSE and RTS-TH-IL CSE at 60 °C at 0.1 mA cm^−2^ @ 0.1 mAh cm^−2^: (a) Zoom-in curves of 1096 h–1104 h; (b) Performances of Li//Li symmetric cells with RTS-TH CSE and RTS-TH-IL CSE at different current densities to determine critical current density with a stripping/plating period of 15 mins; Figure S5: Chronoamperometry polarization curve and the impedance spectra before and after polarization of (a) Li/RTS-TH-IL CSE/Li cell and (b) Li/RTS-TH CSE/Li cell; Figure S6: Galvanostatic charge/discharge curves of LiFePO_4_//Li cells with different CSEs of different cycles (a) in situ RTS-TH-IL CSE; (b) in situ RTS-TH CSE/Li cell; (c) ex situ RTS-TH-IL CSE; Table S1: Ionic conductivities of RTS-TH-IL CSE at different temperatures; Table S2: Ionic conductivities of RTS-TH CSE at different temperatures.

## References

[1] Tseng Y.-C., Hsiang S.-H., Lee T.-Y., Teng H.-S., Jan J.-S., Thein K. (2021). In-situ Polymerized Electrolytes with Fully Cross-Linked Networks Boosting High Ionic Conductivity and Capacity Retention for Lithium Ion Batteries. ACS Appl. Energy Mater..

[2] Lv F., Wang Z.-Y., Shi L.-Y., Zhu J.-F., Edstrom K., Mindemark J., Yuan S. (2019). Challenges and development of composite solid-state electrolytes for high-performance lithium ion batteries. J. Power Sources.

[3] Gao X.-Z., Yuan W., Yang Y., Wu Y.-P., Wang C., Wu X.-Y., Zhang X.-Q., Yuan Y.-H., Tang Y., Chen Y. (2022). High-Performance and Highly Safe Solvate Ionic Liquid-Based Gel Polymer Electrolyte by Rapid UV-Curing for Lithium-Ion Batteries. ACS Appl. Mater. Interfaces.

[4] Kim J., Choi Y.-G., Ahn Y., Kim D., Park J.-H. (2021). Optimized ion-conductive pathway in UV-cured solid polymer electrolytes for all-solid lithium/sodium ion batteries. J. Membr. Sci..

[5] Zeng X.-X., Yin Y.-Y., Li N.-W., Du W.-C., Guo Y.-G., Wan L.-J. (2016). Reshaping Lithium Plating/Stripping Behavior via Bifunctional Polymer Electrolyte for Room-Temperature Solid Li Metal Batteries. J. Am. Chem. Soc..

[6] He L.-C., Ye H.-L., Sun Q.-M., Tieu A.-J.-K., Lu L., Liu Z.-S., Adams S. (2023). In-situ curing enables high performance all-solid-state lithium metal batteries based on ultrathin-layer solid electrolytes. Energy Storage Mater..

[7] Li Z., Xie H.-X., Zhang X.-Y., Guo X. (2020). In-situ thermally polymerized solid composite electrolytes with a broad electrochemical window for all-solid-state lithium metal batteries. J. Mater. Chem. A.

[8] Thomas C., Hyun W.-J., Huang H.-C., Zeng D., Hersam M. (2022). Blade-Coatable Hexagonal Boron Nitride Ionogel Electrolytes for Scalable Production of Lithium Metal Batteries. ACS Energy Lett..

[9] Cha J.H., Didwal P.N., Kim J.M., Chang D.R., Park C.-J., Dauskardt R.H., Cui Y. (2020). Poly(ethylene oxide)-based composite solid polymer electrolyte containing Li_7_La_3_Zr_2_O_12_ and poly(ethylene glycol) dimethyl ether. J. Membr. Sci..

[10] Kou W.-J., Lv R.-X., Zuo S.-W., Yang Z.-H., Huang J.-J., Wu W.-J., Wang J.-T. (2021). Hybridizing polymer electrolyte with poly(ethylene glycol) grafted polymer-like quantum dots for all-solid-state lithium batteries. J. Membr. Sci..

[11] He Y.-Y., Li Y., Tong Q.-S., Zhang J.-D., Weng J.-Z., Zhu M.-Q. (2021). Highly Conductive and Thermostable Grafted Polyrotaxane/Ceramic Hybrid Polymer Electrolyte for Solid-State Lithium-Metal Batteries. ACS Appl. Mater. Interfaces.

[12] Nguyen Q.-H., Luu V.-T., Nguyen H.-L., Lee Y.-W., Cho Y.-H., Kim S.-Y., Jun Y.-S., Ahn W. (2020). Li_7_La_3_Zr_2_O_12_ Garnet Solid Polymer Electrolyte for Highly Stable All-Solid-State Batteries. Front. Chem..

[13] Sun J.-Q., He C.-H., Yao X.-M., Song A.-Q., Li Y.-G., Zhang Q.-H., Hou C.-Y., Shi Q.-W., Wang H.-Z. (2020). Hierarchical Composite-Solid-Electrolyte with High Electrochemical Stability and Interfacial Regulation for Boosting Ultra-Stable Lithium Batteries. Adv. Funct. Mater..

[14] Lu X., Wang Y.-M., Xu X.-Y., Yan B.-G., Wu T., Lu L. (2023). Polymer-Based Solid-State Electrolytes for High-Energy-Density Lithium-Ion Batteries-Review. Adv. Energy Mater..

[15] He K.-Q., Cheng S.-H., Hu J.-Y., Zhang Y.-Q., Yang H.-W., Liu Y.-Y., Liao W.-C., Chen D.-Z., Liao C.-Z., Cheng X. (2021). In-situ Intermolecular Interaction in Composite Polymer Electrolyte for Ultralong Life Quasi-Solid-State Lithium Metal Batteries. Angew. Chem. Int. Ed..

[16] Wang H.-H., Li X.-N., Zeng Q.-H., Li Z.-F., Liu Y., Guan J.-Z., Jiang Y.-C., Chen L., Cao Y., Li R.-Z. (2024). A novel hyperbranched polyurethane solid electrolyte for room temperature ultra-long cycling lithium-ion batteries. Energy Storage Mater..

[17] Zhai Y.-F., Hou W.-S., Tao M.-M., Wang Z.-T., Chen Z.-Y., Zeng Z., Liang X., Paoprasert P., Yang Y., Hu N. (2022). Enabling High-Voltage “Superconcentrated Ionogel-in-Ceramic” Hybrid Electrolyte with Ultrahigh Ionic Conductivity and Single Li^+^-Ion Transference Number. Adv. Mater..

[18] Xie Z.-K., Wu Z.-J., An X.-W., Yoshida A., Wang Z.-D., Hao X.-G., Abudula A., Guan G.-Q. (2019). Bifunctional ionic liquid and conducting ceramic co-assisted solid polymer electrolyte membrane for quasi-solid-state lithium metal batteries. J. Membr. Sci..

[19] Wu Q., Yang Y., Ma C.-C., Chen Z., Su Q.-T., Zhu C.-Z., Gao Y., Ma R., Li C.-H. (2021). Flexible Nanocomposite Polymer Electrolyte Based on UV-Cured Polyurethane Acrylate for Lithium Metal Batteries. ACS Sustain. Chem. Eng..

[20] Xi G., Xiao M., Wang S.-J., Han D.-M., Li Y.-N., Meng Y.-Z. (2020). Polymer-Based Solid Electrolytes: Material Selection, Design, and Application. Adv. Funct. Mater..

[21] Liang J.-N., Luo J., Sun Q., Yang X.-F., Li R.-Y., Sun X.-L. (2019). Recent progress on solid-state hybrid electrolytes for solid-state lithium batteries. Energy Storage Mater..

[22] Deng C.-L., Chen N., Hou C.-Y., Liu H.-X., Zhou Z.-M., Chen R.-J. (2021). Enhancing Interfacial Contact in Solid-State Batteries with a Gradient Composite Solid Electrolyte. Small.

[23] Zhou W.-D., Wang S.-F., Li Y.-T., Sen X., Manthiram A., Goodenough J. (2016). Plating a Dendrite-Free Lithium Anode with a Polymer/Ceramic/Polymer Sandwich Electrolyte. J. Am. Chem. Soc..

[24] Duan H., Yin Y.-X., Shi Y., Wang P.-F., Zhang X.-D., Yang C.-P., Shi J.-L., Wen R., Guo Y.-G., Wan L.-J. (2018). Dendrite-Free Li-Metal Battery Enabled by a Thin Asymmetric Solid Electrolyte with Engineered Layers. J. Am. Chem. Soc..

[25] Wang Y.-L., Wang Z.-L., Zhao Y.-Y., Yang X.-T., Xu J., Ye X.-K., Jiang X.-C., Chen Y.-Z., Chen L., Ye D.-Z. (2024). In-situ polymerization of a free-standing and tough gel polymer electrolyte for lithium metal batteries. J. Power Sources.

[26] Qi S.-G., Li S.-L., Zou W.-W., Zhang W.-F., Wang X.-J., Du L., Liu S.-M., Zhao J.-Q. (2022). Enabling Scalable Polymer Electrolyte with Synergetic Ion Conductive Channels via a Two Stage Rheology Tuning UV Polymerization Strategy. Small.

[27] Wu W., Wei Z.-Y., Wang J., Shang J., Wang M., Chi S.-S., Wang Q.-R., Du L.-L., Zhang T., Zheng Z.-J. (2021). Enabling high-energy flexible solid-state lithium ion batteries at room temperature. Chem. Eng. J..

[28] Tan J.-W., Ao X., Dai A., Yuan Y.-F., Zhuo H., Lu H., Zhuang L.-B., Ke Y.-X., Su C.-L., Peng X.-W. (2020). Polycation ionic liquid tailored PEO-based solid polymer electrolytes for high temperature lithium metal batteries. Energy Storage Mater..

[29] Wu F., Wen Z.-Y., Zhao Z.-K., Bi J.-Y., Shang Y.-X., Liang Y.-H., Li L., Chen N., Li Y.-J., Chen R.-J. (2021). Double-network composite solid electrolyte with stable interface for dendrite-free Li metal anode. Energy Storage Mater..

[30] Lin X.-J., Chu C.-C., Li C., Li Z., Zhang T.-T., Chen J.-Y., Liu R.-Q., Li P., Li Y., Zhao J. (2019). A high-performance, solution-processable polymer/ceramic/ionic liquid electrolyte for room temperature solid-state Li metal batteries. Nano Energy.

[31] Yang Y., Wu Q., Wang D., Ma C.-C., Chen Z., Su Q.-T., Zhu C.-Z., Li C.-H. (2020). Ionic liquid enhanced composite solid electrolyte for high-temperature/long-life/dendrite-free lithium metal batteries. J. Membr. Sci..

