# Supplementary material for: Enhanced Interfacial Contact and Lithium-Ion Transport in Ionic Liquid Polymer Electrolyte via In-Situ Electrolyte-Cathode Integration
Source: Molecules. 2025 Jan 18;30(2):395. doi: 10.3390/molecules30020395 (PMC11767284; doi:10.3390/molecules30020395)
Supplement: Supplementary file 1 [file molecules-30-00395-s001.zip › molecules-3429902-supplementary.pdf]

Supplementary Materials

Enhanced Interfacial Contact and Lithium-Ion Transport in Ionic Liquid Polymer Electrolyte via In-Situ Electrolyte-Cathode Integration

Zehua Chen †, Mianrui Li †, Shengguang Qi and Li Du *

Guangdong Provincial Key Laboratory of Fuel Cell Technology, School of Chemistry and Chemical Engineering, South China University of Technology, Guangzhou 510641, China; chenzh48944@163.com (Z.C.); 202410186301@mail.scut.edu.cn (M.L.); samuel_qsg@aliyun.com (S.Q.)

* Correspondence: duli@scut.edu.cn

† These authors contributed equally to this work

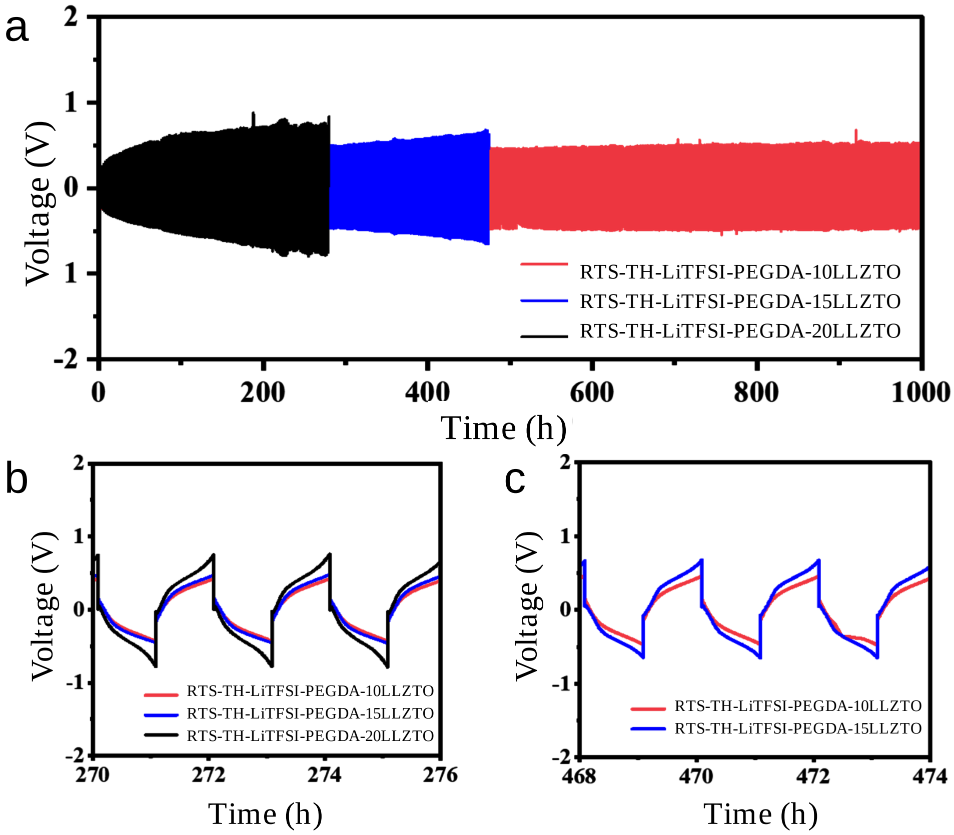

Figure S1. (a) Galvanostatic charge-discharge cycling curves of Li//Li symmetric cells with different LLZTO mass ratio in RTS-TH-LiTFSI-PEGDA-XLLZTO at 60 °C at 0.1 mA cm^{-2} @ 0.1 mAh cm^{-2}; (b) Zoom-in curves at 270 h–276 h in (a); (c) Zoom-in curves at 468 h–474 h in (a).

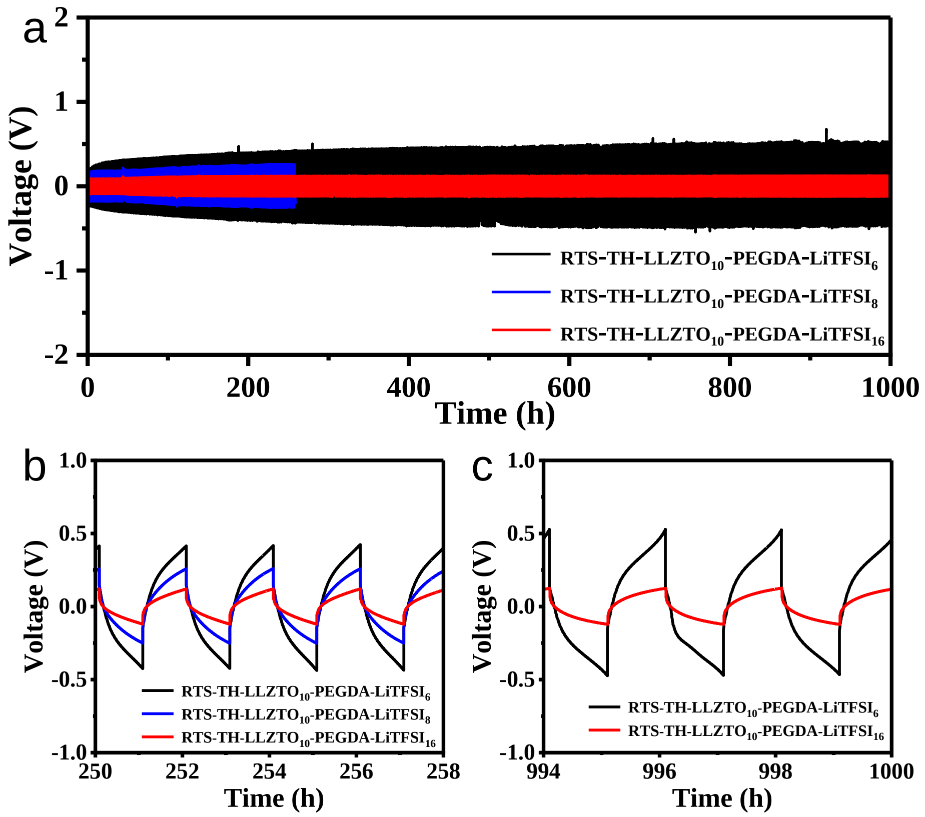

Figure S2. (a) Galvanostatic charge-discharge cycling curves of Li//Li symmetric cells with different EO:Li^+ ratio in RTS-TH-$LLZTO_{10}$-PEGDA-$LiTFSI_x$ at 60 °C at 0.1 mA cm^{-2} @ 0.1 mAh cm^{-2}; (b) Zoom-in curves at 250 h–258 h in (a); (c) Zoom-in curves at 994 h–1000 h in (a).

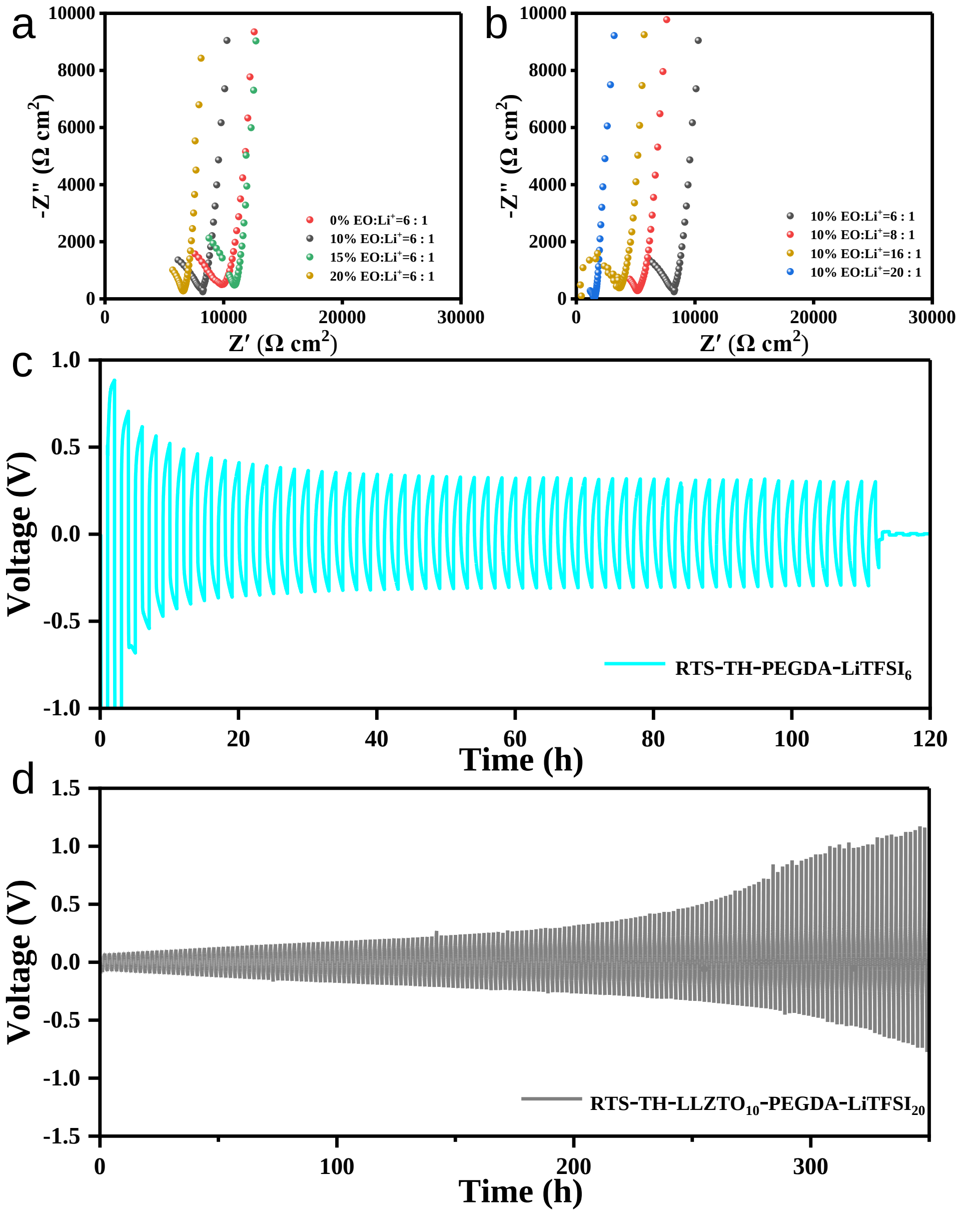

Figure S3. (a) Nyquist curves of RTS-TH-LLZTO$_x$-PEGDA-LiTFSI$_6$ at 30 °C; (b) Nyquist curves of RTS-TH-LLZTO$_{10}$-PEGDA-LiTFSI$_x$ at 30 °C; (c) Galvanostatic charge-discharge cycling curves of Li//Li symmetric cells with RTS-TH-PEGDA-LiTFSI$_6$ at 60 °C at 0.1 mA cm^{-2} @ 0.1 mAh cm^{-2}; (d) Galvanostatic charge-discharge cycling curves of Li//Li symmetric cells with RTS-TH-LLZTO$_{10}$-PEGDA-LiTFSI$_{20}$ at 60 °C at 0.1 mA cm^{-2} @ 0.1 mAh cm^{-2}.

As shown in Figure S1-S3, it should be pointed out that Li//Li symmetric cells employing CSEs with ~10% LLZTO content and EO/Li^+ ratio maintained to be ~16 exhibit lower interfacial impedance and excellent electrochemical performance with stable cycling.

Table S1. Ionic conductivities of RTS-TH-IL CSE at different temperatures

Temperature	30 ℃	40 ℃	50 ℃	60 ℃	70 ℃	80 ℃
ionic conductivity ($mS\ cm^{-1}$)	0.078	0.166	0.338	0.437	0.646	0.844

Table S2. Ionic conductivities of RTS-TH CSE at different temperatures

Temperature	30 ℃	40 ℃	50 ℃	60 ℃	70 ℃	80 ℃
ionic conductivity ($mS\ cm^{-1}$)	0.016	0.037	0.068	0.120	0.194	0.309

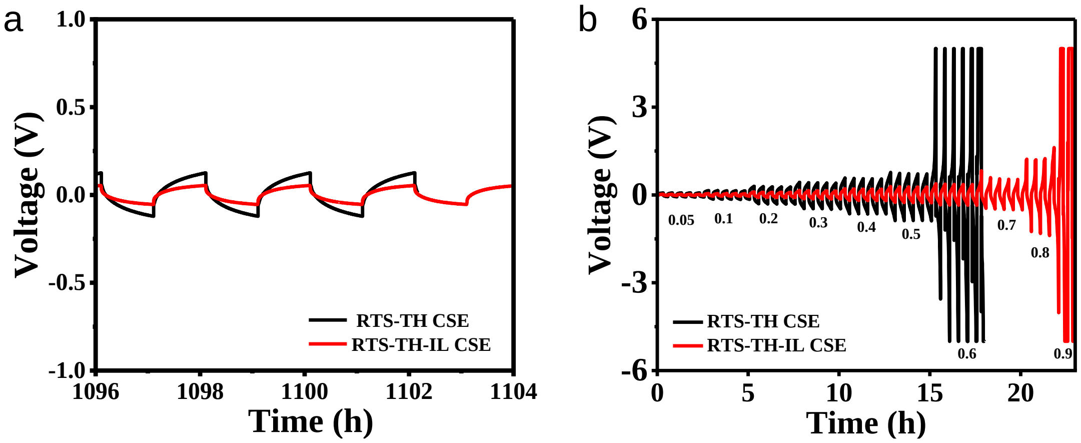

Figure S4. Galvanostatic charge-discharge cycling curves of Li//Li symmetric cells with RTS-TH CSE and RTS-TH-IL CSE at 60 ℃at 0.1 mA cm^{-2} @ 0.1 mAh cm^{-2}: (a) Zoom-in curves of 1096 h –1104 h; (b) Performances of Li//Li symmetric cells with RTS-TH CSE and RTS-TH-IL CSE at different current densities to determine critical current density with a stripping/plating period of 15 mins..

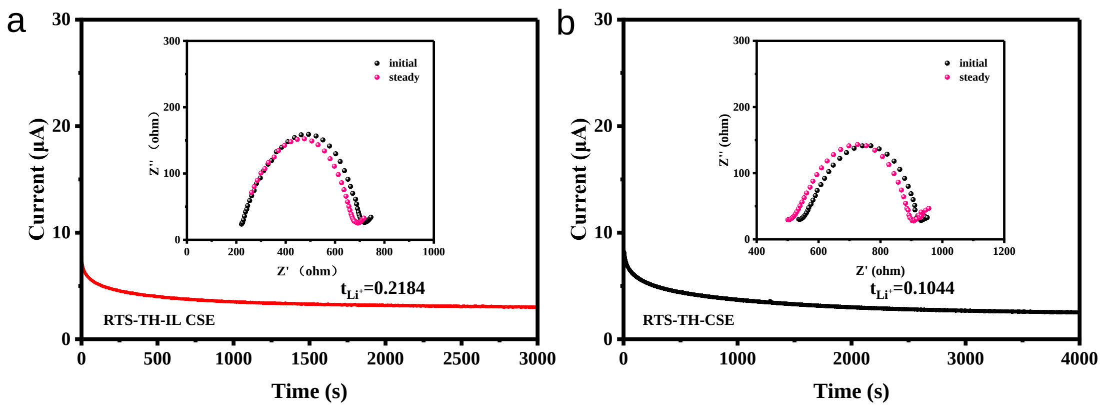

Figure S5. Chronoamperometry polarization curve and the impedance spectra before and after polarization of (a) Li/RTS-TH-IL CSE/Li cell and (b) Li/RTS-TH CSE/Li cell.

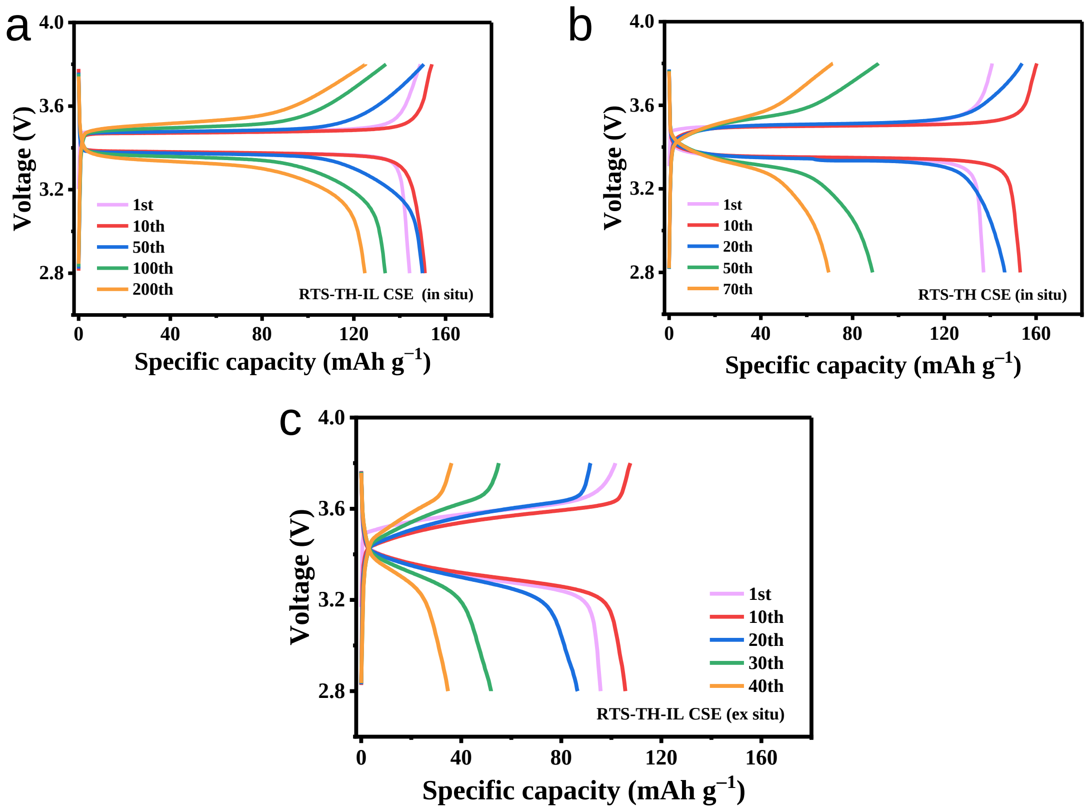

Figure S6. Galvanostatic charge/discharge curves of $LiFePO_4$//Li cells with different CSEs of different cycles (a) in situ RTS-TH-IL CSE ; (b) in situ RTS-TH CSE/Li cell; (c) ex situ RTS-TH-IL CSE.
